# Association Between Pre-diagnostic Dietary Supplements Intake and Ovarian Cancer Survival: Findings From a Prospective Cohort Study in Chinese Women

**DOI:** 10.3389/fnut.2021.758178

**Published:** 2021-12-14

**Authors:** Jia-Hui Gu, Ting-Ting Gong, Qi-Jun Wu, Fang-Hua Liu, Zhao-Yan Wen, Chang Gao, Yi-Fan Wei, Zhuo Yang

**Affiliations:** ^1^Department of Obstetrics and Gynecology, Shengjing Hospital of China Medical University, Shenyang, China; ^2^Department of Clinical Epidemiology, Shengjing Hospital of China Medical University, Shenyang, China; ^3^Clinical Research Center, Shengjing Hospital of China Medical University, Shenyang, China; ^4^Department of Gynecology, Cancer Hospital of China Medical University, Liaoning Cancer Hospital & Institute, Shenyang, China

**Keywords:** cohort, dietary supplements, mortality, ovarian cancer, prognosis, survival

## Abstract

**Background:** As a result of a limited number of studies and inconsistent findings, there remains uncertainty in whether pre-diagnostic dietary supplements intake affects survival after ovarian cancer (OC) diagnosis.

**Methods:** The association between pre-diagnostic dietary supplements intake and all-cause OC mortality was examined in the OC follow-up study, which included a hospital-based cohort (*n* = 703) of Chinese women diagnosed with OC between 2015 and 2020. Pre-diagnostic dietary supplements information was collected using self-administered questionnaires. Deaths were ascertained up to March 31, 2021, *via* death registry linkage. Cox proportional hazards were used to estimate hazard ratios (HR) and 95% confidence intervals (CI) for the aforementioned association.

**Results:** A total of 130 women died during the median follow-up of 37.2 months (interquartile: 24.7–50.2 months). We found no evidence that any pre-diagnostic dietary supplements intake compared with never is associated with OC survival (HR = 0.75, 95%CI: 0.47–1.18). Furthermore, our study suggested no association for ever supplements intakes of vitamin A (HR = 0.48, 95%CI: 0.07–3.46), vitamin C (HR = 0.64, 95%CI: 0.27–1.54), vitamin D (HR = 1.19, 95%CI: 0.28–5.03), vitamin E (HR = 0.47, 95%CI: 0.06–3.87), multivitamin (HR = 0.49, 95%CI: 0.14–1.67), calcium (HR = 0.96, 95%CI: 0.53–1.72), and fish oil/DHA (HR = 0.31, 95%CI: 0.04–2.37) with OC survival. Interestingly, we only found a detrimental effect of vitamin B supplementation intake (HR = 3.78, 95%CI: 1.33–0.69) on OC survival.

**Conclusions:** We found no evidence that any pre-diagnostic dietary supplements intake is associated with OC survival. Considering lower exposure of dietary supplements before OC diagnosis in the present study, further studies are warranted to confirm these findings.

## Introduction

Ovarian cancer (OC) is one of the most common gynecologic cancers, though it occupies third place in mortality, only after cervical and uterine cancer, this disease had the highest mortality rate among gynecologic cancers (1). Due to the lack of early specific symptoms, most cases are diagnosed at a more advanced disease stage. In recent years, the five-year survival rate has increased, however, it was still <50% in China (2). Previous studies suggested that there are different outcomes even among patients with similar characteristics who receive the same treatment (3, 4), indicating there may be factors in addition to non-modifiable cancer characteristics are involved. Therefore, identifying modifiable lifestyle factors that enhance OC survival is worthy of attention. The available pieces of the literature suggest a possible link between dietary and nutrient intake and the incidence and prognosis of OC. For example, a recent umbrella review of 22 systematic reviews and meta-analyses indicates that consumption of black tea or calcium is inversely associated with OC risk, whereas intake of skim/low-fat milk or lactose is positively associated with OC risk (5). Two prospective cohort studies suggest pre-diagnosis consumption of total fruits and vegetables, fish, and fiber can improve survival, while the higher glycemic index and pre-diagnosis meat intake can lead to worse survival (6, 7). Dietary supplement, as a health care product, can supplement nutrients that are insufficient in the human diet, and may also provide a modifiable influence on OC through several mechanisms, such as reducing DNA oxidative damage (8), regulating cell differentiation and apoptosis (9), improving immune function (10), and affecting DNA synthesis and methylation (11).

The mechanisms that drive OC are not fully and clearly understood, but may include elevated circulating gonadotropins, sex-steroid hormones, inflammatory cytokines, and altered glucose homeostasis (12–15). Insulin-like growth factor-I increased the bioavailability of sex steroids and ovarian secretion of androgens (16, 17). Higher circulating estrogen levels may stimulate OC proliferation, resulting in faster growth of metastatic tissue (18). High serum levels of inflammatory markers like tumor Necrosis Factor α, C-reactive protein, interleukin (IL)-4, and IL-6 may indicate that OC progression is associated with poor survival (14, 19). Previous studies reported dietary supplements that presented protection against chronic, low-grade inflammation, and oxidative stress, both of which are suggested to be involved in the onset and progression of cancer (20). A review concluded that whether supplements such as multivitamins or botanicals will convey any benefits to cancer survivors is unclear (21). For example, antioxidant dietary supplements including vitamin A, vitamin C, and vitamin E might reduce DNA damage caused by oxidative stress and increase immune function, regulating cell differentiation and apoptosis to affect OC cells (8, 9, 22). Vitamin B supplements like vitamins B2, B6, and B12 mainly acted as co-factors of methylation, and folic acid might affect DNA synthesis and methylation (11). Calcium supplements might reduce the proliferation of cancer cells by regulating intracellular calcium levels by regulating levels of parathyroid hormone and related proteins (23, 24). The number of randomized controlled trials of dietary supplements for cancer patients conducted to date is very limited, and the safety and effectiveness of dietary supplements have been unclear. Several previous epidemiological studies have reported that consumption of dietary supplements could reduce the risk of OC, including supplements of selenium, vitamin C, vitamin E, β-carotene, calcium, and multivitamin (24–28). However, the evidence between pre-diagnosis dietary supplement intake and OC survival was sparse and inconsistent. Only three observational studies have assessed pre-diagnostic dietary supplements intake and their association with OC survival. For example, a cohort study carried out by Jeffreys et al. (29) demonstrated vitamin D supplements consumption is not associated with survival among women with cancer (including OC women). However, this study failed to adjust for key clinical determinants of survival, like the stage of the disease. An Australian study suggested that pre-diagnostic β-carotene supplements, vitamin C supplements, vitamin E supplements, and multivitamins intake were not related to survival after OC diagnosis (30). Another Australian study conducted by Playdon et al. (7) also observed similar results for the association between vitamin B supplements, vitamin E supplements, and multivitamins intake and survival for patients with OC. In addition, Playdon et al. also reported that vitamin C supplements intake was associated with poor survival during the first 5 years after diagnosis. The aforementioned studies did not completely explore a diverse array of dietary supplements and OC prognosis.

Because of the limited data on these associations, we sought to investigate whether pre-diagnosis dietary supplements intake was associated with OC survival in China. Of note, we evaluated more dietary supplements than previous studies, including vitamin A, vitamin B, vitamin C, vitamin D, vitamin E, multivitamin, calcium, iron, zinc, fish oil/DHA, and ginseng in the present study. The significance of the study is that ours is the first to comprehensively and systematically analyze the relationship between a diverse array of dietary supplements and OC prognosis with adjusting more prognosis-related factors in the well-designed prospective cohort study in China.

## Methods

### Study Population

The ovarian cancer follow-up study (OOPS) is a prospective longitudinal cohort study of women with OC with the purpose of evaluating the relationship between lifestyle and outcomes. Women met the following criteria: (1) patients recruited during the baseline survey were between 18 and 79 years old; (2) patients with the incident, histologically confirmed OC; (3) Their follow-up and medical treatment were conducted at the gynecological oncology ward at Shengjing Hospital of China Medical University, Shenyang, China after 2015; and (4) all patients were able to answer the epidemiological questionnaire. The study was approved by the Institutional Review Board of the Ethics Committee of Shengjing Hospital of China Medical University. Information collection on long-term outcomes is still ongoing. After excluding participants who refused to participate or did not return the study questionnaire, 744 out of 853 women consented to participate. Participants were excluded if they reported significantly abnormal caloric intake (<500 or >3,500 calories per day) (*n* = 17) (31) or left 11 (10.00%) or more food items blank (*n* = 24). The resulting cohort included 703 eligible patients (Supplementary Figure 1).

### Data Collection

Demographic characteristics and lifestyle factors were collected in the baseline interview using self-administered questionnaires. The questionnaires include educational level, monthly household income, physical activity (work, commuting, household chores, and leisure-time exercise), smoking status, alcohol drinking, and tea drinking. The anthropometric measurements, such as height and body weight, were measured by trained staff following a standard protocol and using height and body weight, we further calculated body mass index (BMI), which is calculated as weight in kilograms/height in m^2^. Clinical data regarding age at diagnosis, histological type (serous or non-serous), histopathologic grade (well, moderate, and poorly differentiated), International Federation of Gynecology and Obstetrics (FIGO) stage (I–II, III–IV, and unknown) (32), residual lesions, and comorbidities were abstracted from the electronic medical records of the Shengjing hospital information system.

### Dietary Supplements Assessment

Well-trained interviewers conducted face-to-face interviews using a self-administered questionnaire to gather information on dietary supplements. Participants were asked whether they used any type of supplements for longer than 2 months during the past year prior to OC diagnosis and to identify the types of dietary supplements that were taken. Dietary supplements include vitamin A, vitamin B, vitamin C, vitamin D, vitamin E, multi-vitamin, calcium, iron, zinc, fish oil/DHA, and ginseng. Quality control was achieved through the double entry of each questionnaire and manual adjudication.

### Follow-up and Outcome

Information on the vital status of OOPS participants was ascertained based on passive and active modes. In the passive mode, the follow-up was carried out by linkage to the Liaoning Providence Center for Disease Control and Prevention every 6 months. In addition, clinical specialists gathered patient medical data from the information system at Shengjing Hospital every 6 months after the patient finished the baseline survey. This time lapse allowed for definitive staging, pathology evaluation, diagnosis determination, and initial treatment to be completed. In the active model, patients with OC were followed up by telephone contact. In the current analysis, the outcome was focused on death from any cause. Survival time was defined as the interval between histologic diagnosis and date of death from any cause or the date of last follow-up (March 31, 2021) for patients who were still alive.

### Statistical Analysis

We used the Kolmogorov-Smirnov statistic to test the normality for all continuous variables. To compare socio-demographic and clinical characteristics between the two groups, Student's *t*-test or the Kruskal–Wallis test was applied for continuous variables and the Chi-square test for categorical variables. The drawing of crude survival curve and the estimation of crude overall survival probabilities were based on the Kaplan–Meier technique. Cox proportional hazards models were used to estimate hazard ratios (HR) and 95% confidence intervals (CI), which were used for the association of pre-consumption of dietary supplements with overall OC survival after estimating the proportional hazards assumption by adding an interaction term between each activity variable and log survival time. All the variables in this analysis have satisfied the conditions (all *p* > 0.05).

Dietary supplement intake was divided into intake groups and non-intake groups. The non-intake groups served as the reference groups. In order to better control the influence of confounding factors, we used the following three criteria to select covariates: (1) clinical significance; (2) previous studies; and (3) degree of correlation with the exposure. The first model only adjusted for age at diagnosis (<50, ≥50 years), and the second model further adjusted for body mass index (continuous, kg/m^2^), physical activity (continuous, MET/hours/days), diet change (yes or no), dietary pattern (derived from principal components for factor analysis) (33), total energy intake (continuous, kcal), education (junior secondary or below, senior high school/technical secondary school, and junior college/university or above), FIGO stage (I–II, III–IV, and unknown), histological type (serous, non-serous), histopathologic grade (well, moderate, and poorly differentiated), residual lesions (none, <1, ≥1 cm), menopausal status (yes or no), parity (≤ 1, ≥2), oral contraceptive use (yes or no), and smoke status (yes or no). Of these, physical activity was assessed based on a previous study (34). Briefly, participants were asked the usual type and duration of activities related to work, commuting, household chores, and leisure-time exercise during the past 12 months in our study (34). We further used metabolic equivalent tasks (METs) from the 2011 update (35) of a major compendium of physical activities to calculate the amount of physical activity. For a dietary change, participants were asked whether they have changed their diet recently. Dietary pattern was defined by the 2015 Dietary Guidelines Advisory Committee as “the quantities, proportions, variety or combination of different foods, drinks, and nutrients (when available) in diets and the frequency with which they are consumed” (36). In addition, a study has indicated that dietary patterns could be used as a covariate to determine whether the effect of nutrients was independent of the overall dietary pattern (37). In the present analysis, we used 111 food items included in food frequency questionnaire (FFQ) to perform factor analysis. The correlation matrix among the 111 food items was visually and statistically examined to justify undertaking factor analysis. Next, we kept meaningful components from the total number of extracted nutrient patterns, and the number of meaningful components depended mainly on the assessment of scree plots and components' interpretability. Finally, we abstracted 5 factors in our analysis. All analyses were conducted using SAS statistical software (version 9.4; SAS Institute, Cary, NC, USA). All statistical tests were two-sided and statistical significance was defined as *p* < 0.05.

## Results

The distribution of general baseline characteristics among patients with OC according to dietary supplements intake is presented in Table 1. The median (interquartile) age at diagnosis and follow-up time among 703 patients with OC was 53.0 (48.0–60.0) years and 37.2 (24.7–50.2) months, respectively, with no significant difference when classified according to the intake of dietary supplements. However, participants consuming any dietary supplements had a significantly higher intake of meat, dairy products, beans and bean products, eggs, and total energy than participants who did not consume dietary supplements. Furthermore, the proportion of smokers in patients consuming any dietary supplements was significantly lower than the proportion in patients consuming none. In terms of other characteristics in Table 1, we found no significant differences between the two groups. Table 2 shows that non-serous histological subtype, a larger volume of residual disease, and later-stage disease were significantly associated with higher mortality among OC survivors in this study.

**Table 1:** General characteristics of patients with ovarian cancer (OC) according to dietary supplements intake (*N* = 703).

**Variables**	**Any supplements intake**		***P* value**
	**Yes**	**No**	
**No. of patients**	150	553	
**Age at diagnosis (years), Median (IQR)**	54.00 (47.00–61.00)	53.00 (48.00–60.00)	0.66
**Follow-up time (months), Median (IQR)**	33.95 (19.77–47.87)	30.60 (20.10–46.63)	0.46
**Body mass index (kg/m** ^**2**^ **), Median (IQR)**	22.85 (20.40–24.70)	23.30 (21.00–25.10)	0.07
**Physical activity (MET/hours/days), Median (IQR)**	13.85 (7.30–22.90)	14.10 (6.30–22.00)	0.83
**Diet intake (Mean** **±** **SD)**			
Total energy (kcal/d)	1584.58 ± 572.89	1420.81 ± 542.30	<0.05
Meat (g/day)	40.69 ± 31.52	35.21 ± 28.81	<0.05
Dairy products (g/day)	102.28 ± 126.41	79.05 ± 109.00	<0.05
Eggs (g/day)	42.60 ± 28.10	36.44 ± 26.74	<0.05
Fish and seafood (g/day)	32.13 ± 28.01	27.54± 30.85	0.10
Beans and bean products (g/day)	101.15 ± 81.45	80.96 ± 77.13	<0.05
Vegetables (g/day)	225.66 ± 119.92	211.12 ± 122.12	0.19
Fruits (g/day)	209.63 ± 152.07	190.57 ± 159.22	0.19
**Diet change (** ***n*** **, %)**			0.08
No	106 (70.67)	429 (77.58)	
Yes	44 (29.33)	124 (22.42)	
**Smoke status (** ***n*** **, %)**			<0.05
No	142 (94.67)	493 (89.15)	
Yes	8 (5.33)	60 (10.85)	
**Alcohol intake (** ***n*** **, %)**			0.96
No	118 (78.67)	436 (78.84)	
Yes	32 (21.33)	117 (21.16)	
**Tea drinking (** ***n*** **, %)**			0.30
No	107 (71.33)	370 (66.91)	
Yes	43 (28.67)	183 (33.09)	
**Menopausal status (** ***n*** **, %)**			0.62
No	44 (29.33)	151 (27.31)	
Yes	106 (70.67)	402 (72.69)	
**Parity (** ***n*** **, %)**			0.57
≤ 1	105 (70.00)	400 (72.33)	
≥ 2	45 (30.00)	153 (27.67)	
**Educational level (** ***n*** **, %)**			0.86
Junior secondary or below	81 (54.00)	294 (53.16)	
Senior high school/technical secondary school	29 (19.33)	118 (21.34)	
Junior college/university or above	40 (26.67)	141 (25.50)	
**Income per month (Yuan), (** ***n*** **, %)**			0.18
<5000	80 (53.33)	341 (61.66)	
5000 to <10000	49 (32.67)	145 (26.22)	
≥10000	21 (14.00)	67 (12.12)	

*IQR, interquartile range; MET, metabolic equivalent task; SD, standard deviation*.

**Table 2:** Selected clinical characteristics and associations with all-cause mortality among women diagnosed with ovarian cancer (*N* = 703).

**Characteristic**	**No. of deaths/total (%)**	**Crude HR (95% CI)**	***P* value**	**Adjusted HR^**a**^ (95% CI)**	***P* value**
**Age at diagnosis**					
≤ 50	45/258 (17.44)	1.00 (ref)		1.00 (ref)	
>50	85/445 (19.10)	1.18 (0.82–1.70)	0.37	1.24 (0.85–1.79)	0.26
**Histological type**					
Serous	92/479 (19.21)	1.00 (ref)		1.00 (ref)	
Non-serous	38/224 (16. 96)	0.87 (0.59–1.27)	0.47	1.71 (1.11–2.66)	<0.05
**Histopathologic grade**					
Well differentiated	5/56 (8.93)	1.00 (ref)		1.00 (ref)	
Moderately differentiated	7/48 (14.58)	1.44 (0.46–4.57)	0.53	1.12 (0.35–3.57)	0.85
Poorly differentiated	118/599 (19.70)	2.32 (0.95–5.67)	0.07 00	1.76 (0.70–4.43)	0.23
**FIGO stage**					
I–II	41/342 (11.99)	1.00 (ref)		1.00 (ref)	
III–IV	89/338 (26.33)	2.75 (1.89–4.00)	<0.05	2.54 (1.65–3.91)	<0.05
**Residual lesions**					
No	82/553 (14.83)	1.00 (ref)		1.00 (ref)	
<1 cm	31/106 (29.25)	2.22 (1.47–3.36)	<0.05	1.73 (1.11–2.68)	<0.05
≥1 cm	17/44 (38.64)	3.18 (1.89–5.37)	<0.05	2.41 (1.39–4.16)	<0.05
**Comorbidities**					
No	74/393 (18.83)	1.00 (ref)		1.00 (ref)	
Yes	56/310 (18.06)	0.82 (0.58–1.16)	0.27	0.97 (0.68–1.38)	0.86

a *Mutually adjusted for all other variables listed in the table.*

*CI, confidence interval; HR, hazard ratio; ref, reference*.

**Figure 1:**
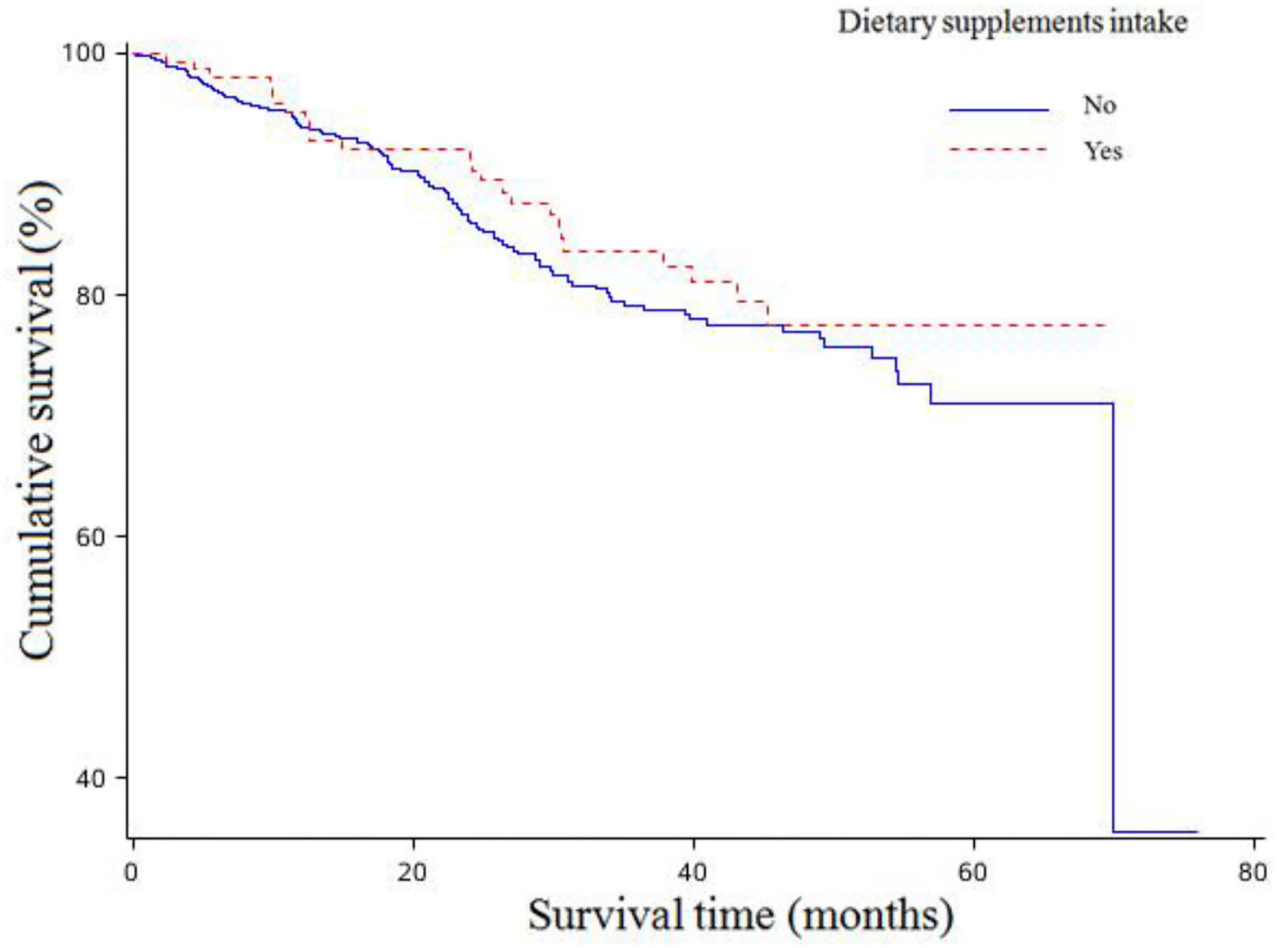
Kaplan-Meier survival curves for dietary supplements intake.

The associations between dietary supplements intake and overall survival of OC are shown in Table 3. There were 24 (16.00%) of 150 patients consuming any dietary supplements who died during the follow-up time compared to 106 women (19.17%) who still survived among the 533 patients consuming none. There were no statistically significant associations between any dietary supplements intake and OC survival (Figure 1). In addition, no significant associations were found between pre-diagnosis consumption of all kinds of dietary supplements and OC survival in the age-adjusted model. However, after adjustments of more confounding factors, we observed pre-diagnosis consumption of vitamin B was associated with worse survival (HR = 3.78, 95%CI: 1.33–10.69) (Supplementary Figure 2).

**Table 3:** Hazard ratio (95% CI) for overall survival among patients with OC according to different dietary supplements intake.

**Vitamin Supplement intake**	**No**	**Yes**	***P* value**
**Any supplements**			
No. of deaths/total (%)	106/553 (19.17)	24/150 (16.00)	
Model 1^a^ HR (95% CI)	1.00 (ref)	0.82 (0.53–1.28)	0.37
Model 2^b^ HR (95% CI)	1.00 (ref)	0.75 (0.47–1.18)	0.22
**Vitamin A**			
No. of deaths/total (%)	129/692 (18.64)	1/11 (9.09)	
Model 1^a^ HR (95% CI)	1.00 (ref)	0.42 (0.06–2.98)	0.38
Model 2^b^ HR (95% CI)	1.00 (ref)	0.48 (0.07–3.46)	0.46
**Vitamin B**			
No. of deaths/total (%)	126/691 (18.23)	4/12 (33.33)	
Model 1^a^ HR (95% CI)	1.00 (ref)	2.19 (0.81–5.94)	0.12
Model 2^b^ HR (95% CI)	1.00 (ref)	3.78 (1.33–10.96)	<0.05
**Vitamin C**			
No. of deaths/total (%)	124/662 (18.73)	6/41 (14.63)	
Model 1^a^ HR (95% CI)	1.00 (ref)	0.79 (0.35–1.79)	0.57
Model 2^b^ HR (95% CI)	1.00 (ref)	0.64 (0.27–1.54)	0.32
**Vitamin D**			
No. of deaths/total (%)	128/694 (18.44)	2/9 (22.22)	
Model 1^a^ HR (95% CI)	1.00 (ref)	1.27 (0.31–5.12)	0.74
Model 2^b^ HR (95% CI)	1.00 (ref)	1.19 (0.28–5.03)	0.82
**Vitamin E**			
No. of deaths/total (%)	129/694 (18.59)	1/9 (11.11)	
Model 1^a^ HR (95% CI)	1.00 (ref)	0.69 (0.10–4.91)	0.71
Model 2^b^ HR (95% CI)	1.00 (ref)	0.47 (0.06–3.87)	0.48
**Multivitamin**			
No. of deaths/total (%)	127/672 (18.90)	3/31 (9.68)	
Model 1^a^ HR (95% CI)	1.00 (ref)	0.47 (0.15–1.48)	0.20
Model 2^b^ HR (95% CI)	1.00 (ref)	0.49 (0.14–1.67)	0.26
**Calcium**			
No. of deaths/total (%)	117/637 (18.37)	13/66 (19.70)	
Model 1^a^ HR (95% CI)	1.00 (ref)	1.11 (0.63–1.97)	0.72
Model 2^b^ HR (95% CI)	1.00 (ref)	0.96 (0.53–1.72)	0.88
**Iron**			
No. of deaths/total (%)	130/698 (18.62)	0/5 (0.00)	
Model 1^a^ HR (95% CI)	1.00 (ref)	–	
Model 2^b^ HR (95% CI)	1.00 (ref)	–	
**Zinc**			
No. of deaths/total (%)	130/700 (18.57)	0/3 (0.00)	
Model 1^a^ HR (95% CI)	1.00 (ref)	–	
Model 2^b^ HR (95% CI)	1.00 (ref)	–	
**Fish oil/DHA**			
No. of deaths/total (%)	129/685 (18.83)	1/18 (5.56)	
Model 1^a^ HR (95% CI)	1.00 (ref)	0.26 (0.04–1.83)	0.17
Model 2^b^ HR (95% CI)	1.00 (ref)	0.31 (0.04–2.37)	0.26
**Ginseng**			
No. of deaths/total (%)	130/702 (18.52)	0/1 (0.00)	
Model 1^a^ HR (95% CI)	1.00 (ref)	–	
Model 2^b^ HR (95% CI)	1.00 (ref)	–	

*CI, confidence interval; HR, hazard ratio; ref, reference.*

a *Model 1 adjusted for age at diagnosis.*

b *Model 2 same as Model 1 and further adjusted for body mass index, diet change, dietary pattern, total energy intake, education, FIGO stage, histological type, histopathologic grade, menopausal status, parity, oral contraceptive use, physical activity, residual lesions, and smoke status*.

## Discussion

Our findings from a Chinese hospital-based sample of women diagnosed with OC suggest no association between any dietary supplement intake and survival after diagnosis. Furthermore, we observed no association for ever intakes of vitamin A, vitamin C, vitamin D, vitamin E, multivitamin, calcium, and fish oil/DHA supplementation with OC survival. While ever consumption of vitamin B supplements was associated with worse OC survival after controlling for multiple confounding factors.

### Comparison With Other Studies

Several studies have assessed the associations of dietary supplement intake with the incidence and prognosis of OC. Most of them focused on the impact of dietary supplements on the risk of OC. For example, Pan et al. (26) found that consuming supplements of vitamin E and beta-carotene for ≥10 years was inversely associated with the risk of OC, whereas consuming supplements of vitamin A, vitamin B, vitamin C, calcium, selenium, and multivitamin was unrelated with OC risk. Thomson et al. (38) suggested that intake of dietary supplements (vitamin A, vitamin C, vitamin E, beta-carotene, and selenium) was not associated with a reduction in OC risk among postmenopausal women. Additionally, Gifkins et al. (39) reported opposite results, in which supplement users had significantly increased OC risk for vitamin A, vitamin C, vitamin E, selenium, and beta-carotene. However, only three studies have investigated the relationship between pre-diagnosis intake of dietary supplements and OC survival, and the results have been inconsistent. In general, the present findings were in line with two previous studies (29, 30). Jeffrey et al. (29) conducted a cohort study recruiting women aged ≥55 years from the UK Clinical Practice Research Datalink (CPRD) and found prescription of vitamin D was not associated with OC survival. An earlier cohort study reported that consuming vitamin E, vitamin C, beta-carotene, or multivitamins from supplements did not have any survival advantage among 609 Australian patients with epithelial OC (30). However, compared with the present findings, results from Playdon et al. (7) were partially inconsistent. Playdon et al. observed that a null association between vitamin B, vitamin E, and multivitamin supplements, and OC mortality, and consumption of vitamin C (above 180 mg per day) was associated with poor survival (HR = 1.36, 95%CI: 1.04–1.78) during the first 5 years but was not associated after 5 years. This inconsistency might be attributed to the different proportions of participants consuming dietary supplements. The proportions in the study by Playdon et al. (7) (vitamin B: 11.0%; vitamin C: 33.0%) were relatively larger than the present study (vitamin B: 1.7%; vitamin C: 5.8%). Additionally, different adjustments of covariates, in the final analysis, should not be ignored.

There were also some studies that explored the effects of dietary supplements on other cancers risk and mortality. Alsharairi et al. (40) performed a review and suggested caution in recommending long-term, high-dose supplements that contain β-carotene, retinyl palmitate, vitamin E, and B vitamins (B6, B12) for patients with lung cancer, especially current and former smokers. Afroditi et al. (41) indicated a potentially beneficial role of post-diagnosis calcium and vitamin D supplementation on cancer prognosis based on a systematic review and meta-analysis. Findings from a meta-analysis suggested that efforts to achieve circulating levels of 25(OH)D around 54–135 nmol/L may contribute to reducing cancer mortality (42).

### Potential Biological Mechanisms

Different categories of dietary supplements have various underlying biological mechanisms on the prognosis of patients with OC. Antioxidant dietary supplements (vitamin A, vitamin C, vitamin E) might affect OC cells by reducing DNA damage caused by oxidative stress and increasing immune function, regulating cell differentiation and apoptosis (8, 9, 22). Beta-carotene is also a potent antioxidant and can improve immune function (10, 43). Previous evidence has suggested that selenium may provide antioxidant protection and anticancer properties (39, 44). Human studies have indicated that increased selenium could reduce the risk of colorectal, lung, and prostate cancers (45, 46). Moreover, animal studies have indicated that selenium compounds may reduce cancer cell proliferation, inhibit cancer cell growth, and stimulate programmed cancer cell death at high doses (39). Folic acid in vitamin B supplements might affect DNA synthesis and methylation, and vitamins B2, B6, and B12 mainly acted as co-factors of methylation (11). Calcium supplements might reduce the proliferation of colorectal cancer cells by regulating intracellular calcium levels by regulating levels of parathyroid hormone and related proteins, and their effect on OC cells might be related because mucinous tumors of OC usually originate in colorectal cancer (23, 24). Vitamin D played its role mainly through calcium-mediated apoptosis (47).

### Strengthens and Limitations

The current study has some critical points worth being emphasized. This investigation is, to our knowledge, the first study to explore the association between pre-diagnostic dietary supplements intake and mortality among patients with OC in China. A further strength is a prospective design with high participation rates in the baseline survey and a small proportion of loss to follow-up, which minimize the likelihood of recall bias or selection bias. Additionally, detailed information on tumor characteristics, like residual lesions, histological type, and FIGO stage were collected, allowing us to rigorously adjust prognosis-related confounding factors, which provided more credible findings.

However, several limitations should be mentioned. First, dietary supplements intake of patients with OC in our investigation was based on self-report at baseline, and recall bias was unavoidable. Nevertheless, dietary supplements information was acquired by experienced and well-trained investigators, and the participants with higher energy intake (>3,500 kcal) and lower energy intake (<500 kcal) were excluded in our analysis, which reduced the misclassification error due to recall bias. Additionally, based on our questionnaire, we could only collect whether and how long dietary supplements were consumed rather than the intake frequency or intake dosage of dietary supplements. Second, dietary supplements intake among patients with OC was evaluated through a questionnaire survey before diagnosis rather than after diagnosis and was used to replenish micronutrients that are insufficient in the diet for the human body (5). In reality, having a chronic disease like cancer might prompt the initiation of dietary supplement intake (48), therefore, future studies need to focus on the effect of post-diagnostic dietary supplement intake on prognosis among patients with OC and further clarify the aforementioned topic. Third, data reported that 49.00% of healthy adults in the United States had dietary supplement use in the past 30 days from 2007 to 2010 (49), while the proportion of men and women dietary supplements use was 15.07 and 19.71%, respectively, from the Shanghai Men's Health Study and Shanghai Women's Health Study (50). Of note, this proportion in our study was 21.38%. Therefore, the results of our investigation need to be interpreted with caution. Furthermore, due to the low exposure rate of dietary supplements intake in the present study, we failed to perform subgroup analyses stratified by participants' characteristics including BMI and menopausal status. Fourth, we failed to explore the correlation between pre-diagnostic dietary supplement intake and progression-free survival among patients with OC, but progression-free survival might be close to overall survival because of the high mortality rate and short post-progression survival period for OC (51, 52). Fifth, residual confounding is inevitable for any observational study. Though most prognosis-related confounders were comprehensively adjusted in our analysis, the impact of unknown or unmeasured confounders might not be ruled out. Further researches are necessary to rule out the possibility of these problems and to better elucidate the aforementioned association.

## Conclusion

In summary, in this cohort study in women in China, our results do not support any association between consumption of vitamin A, vitamin C, vitamin D, vitamin E, multivitamin, calcium, and fish oil/DHA supplementation and beneficial survival from OC. In addition, future studies are needed to confirm the effects detected for vitamin B supplements on survival from OC. The findings provide some empirical data for consideration for the use of dietary supplements which should be confirmed in the future.

## Data Availability Statement

The raw data supporting the conclusions of this article will be made available by the authors, without undue reservation.

## Ethics Statement

The studies involving human participants were reviewed and approved by Shengjing hospital of China Medical University. The patients/participants provided their written informed consent to participate in this study.

## Author Contributions

T-TG and Q-JW: study design. F-HL, Z-YW, CG, and Y-FW: collection of data. F-HL and Y-FW: analysis of data. J-HG, T-TG, Q-JW, F-HL, Z-YW, CG, and Y-FW: drafting the manuscript. J-HG and ZY revision of the manuscript. All authors have approved the final article.

## Funding

This work was supported by Shenyang Young and Middle-Aged Scientific and Technological Innovation Talents Project (No. RC200499 to ZY) and The Fundamental Research Funds for the Central Universities (No. LD202013 to ZY).

## Conflict of Interest

The authors declare that the research was conducted in the absence of any commercial or financial relationships that could be construed as a potential conflict of interest.

## Publisher's Note

All claims expressed in this article are solely those of the authors and do not necessarily represent those of their affiliated organizations, or those of the publisher, the editors and the reviewers. Any product that may be evaluated in this article, or claim that may be made by its manufacturer, is not guaranteed or endorsed by the publisher.
