# Corrigendum: Association Between Pre-diagnostic Dietary Supplements Intake and Ovarian Cancer Survival: Findings From a Prospective Cohort Study in Chinese Women

**DOI:** 10.3389/fnut.2022.856540

**Published:** 2022-02-10

**Authors:** Jia-Hui Gu, Ting-Ting Gong, Qi-Jun Wu, Fang-Hua Liu, Zhao-Yan Wen, Chang Gao, Yi-Fan Wei, Zhuo Yang

**Affiliations:** ^1^Department of Obstetrics and Gynecology, Shengjing Hospital of China Medical University, Shenyang, China; ^2^Department of Clinical Epidemiology, Shengjing Hospital of China Medical University, Shenyang, China; ^3^Clinical Research Center, Shengjing Hospital of China Medical University, Shenyang, China; ^4^Department of Gynecology, Cancer Hospital of China Medical University, Liaoning Cancer Hospital & Institute, Shenyang, China

**Keywords:** cohort, dietary supplements, mortality, ovarian cancer, prognosis, survival

There is an error in the Funding statement. The author that acquired funding should be Zhuo Yang. The corrected funding statement is below.

This work was supported by Shenyang Young and Middle-Aged Scientific and Technological Innovation Talents Project (No. RC200499 to ZY) and The Fundamental Research Funds for the Central Universities (No. LD202013 to ZY).

The authors apologize for this error and state that this does not change the scientific conclusions of the article in any way. The original article has been updated.

## Publisher's Note

All claims expressed in this article are solely those of the authors and do not necessarily represent those of their affiliated organizations, or those of the publisher, the editors and the reviewers. Any product that may be evaluated in this article, or claim that may be made by its manufacturer, is not guaranteed or endorsed by the publisher.

