# Supplementary material for: Association Between Pre-diagnostic Dietary Supplements Intake and Ovarian Cancer Survival: Findings From a Prospective Cohort Study in Chinese Women
Source: Front Nutr. 2021 Dec 14;8:758178. doi: 10.3389/fnut.2021.758178 (PMC8712326; doi:10.3389/fnut.2021.758178)
Supplement: Supplementary file 1 [file Data_Sheet_1.pdf]

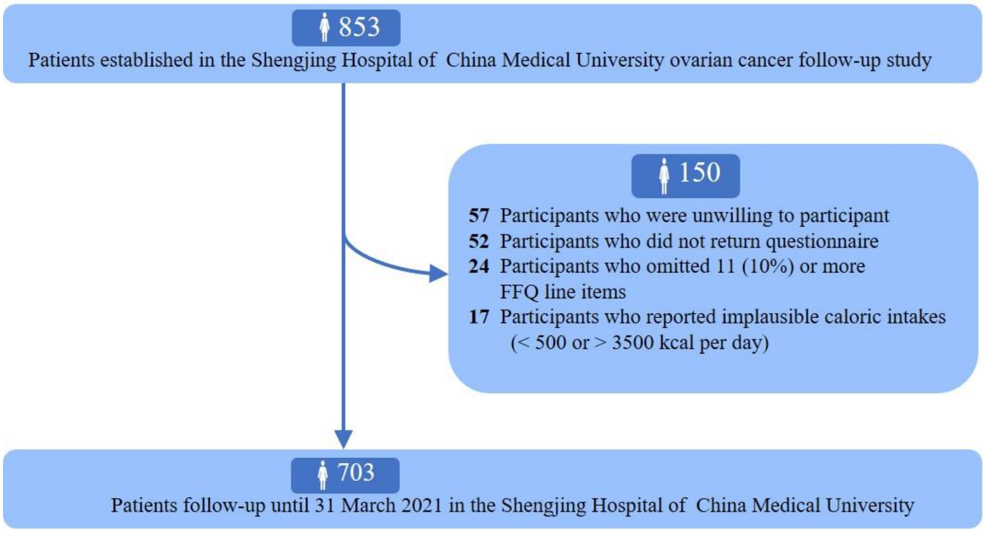


**Supplementary figure 1** Flow of participants through study

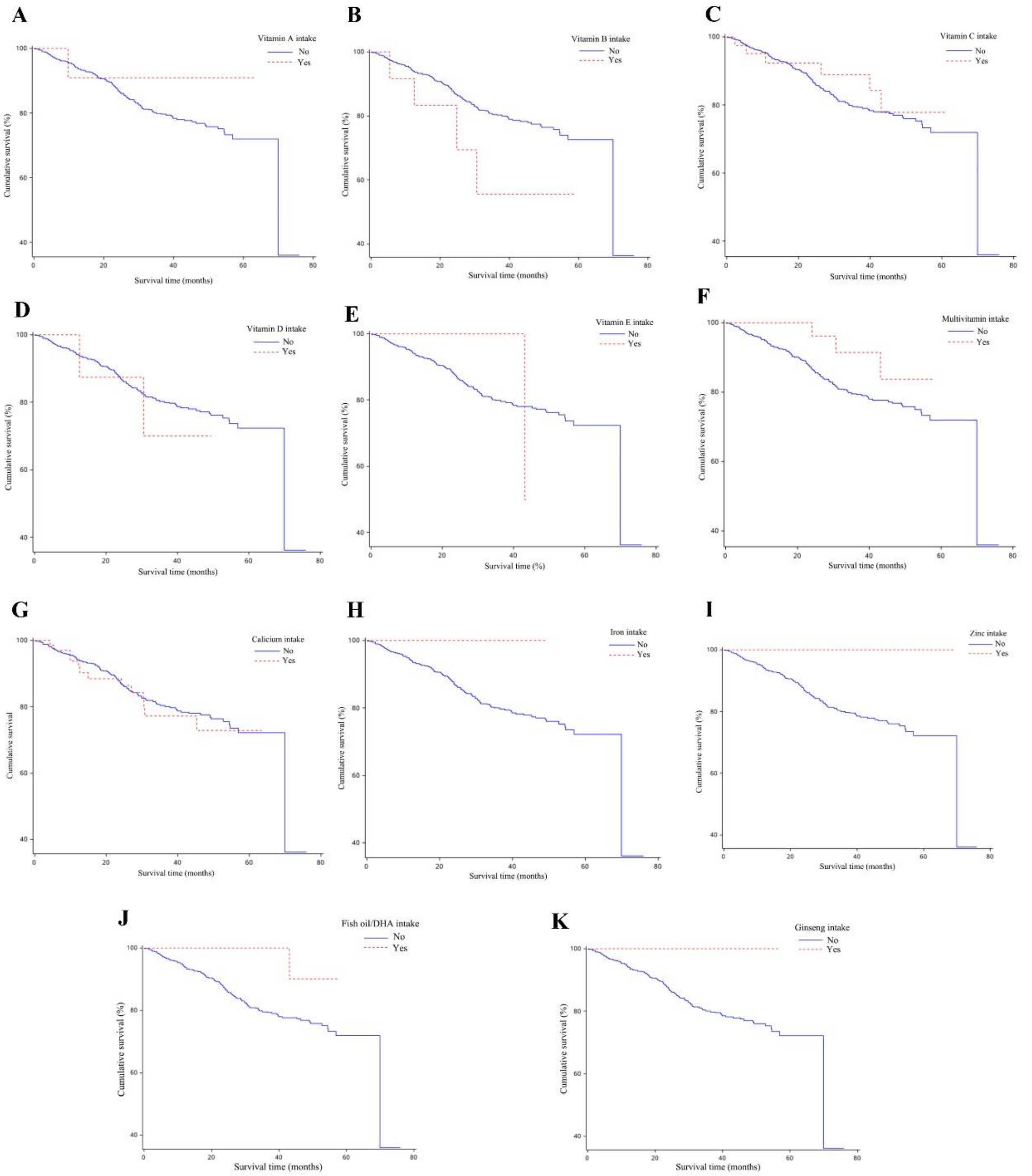


**Supplementary figure 2** Kaplan-Meier curves for Vitamin A (A), Vitamin B (B), Vitamin C (C), Vitamin D (D), Vitamin E (E), Multivitamin (F), Calcium (G), Iron (H), Zinc (I), Fish oil/DHA (J), and Ginseng (K).
